# Continuous Low-dose Epidural Morphine and Ketamine Analgesia Improves Quality of Recovery after Major Lumbar Spine Surgery: A Randomised Controlled Trial

**DOI:** 10.4274/TJAR.2025.251950

**Published:** 2025-12-22

**Authors:** Sailaja Karri, Ramamani Mariappan, Gandham Edmond Jonathan, Thenmozhi Mani, Prasadkanna Prabhakar, Jemimah Samuel, Krishnaprabhu Raju

**Affiliations:** 1 Christian Medical College &amp; Hospital Vellore, Department of Anaesthesia, State of Tamil Nādu, Vellore, India; 2 Christian Medical College Vellore, Department of Neuroanaesthesia, State of Tamil Nādu, India; 3 Christian Medical College Vellore, Ranipet Campus, Department of Neurological Sciences, State of Tamil Nādu, Ranipet, India; 4 Christian Medical College Vellore, Department of Biostatistics, State of Tamil Nādu, Bagayam, India; 5 Christian Medical College Vellore, Department of Anaesthesia, Vellore, India; 6 Christian Medical College Vellore, Department of Nursing, Acute Pain Service, Vellore, India

**Keywords:** Epidural analgesia, ketamine, lumbar spine surgery, postoperative pain, quality of recovery

## Abstract

**Objective:**

The effect of postoperative analgesia on the quality of recovery (QoR) after major lumbar spine surgery is understudied. We hypothesized that continuous epidural morphine and ketamine administration would provide effective analgesia, thereby improving QoR compared to continuous intravenous morphine and ketamine using the QoR-15 questionnaire.

**Methods:**

A total of 40 patients were randomised to receive either continuous low-dose epidural morphine and ketamine via an intraoperatively placed epidural catheter (Group A) or intravenous morphine and ketamine using a patient-controlled analgesia system (Group B) for 48 hours. All patients were anaesthetized using standard anaesthesia drugs. The primary outcome was QoR at 24 and 48 hours after surgery using the QoR-15 questionnaire. The secondary outcomes were pain score at various time points during the first 48 hours, rescue analgesic requirements, ambulation time, length of hospital stay, and patient satisfaction.

**Results:**

Forty patients were recruited (20 in each group), and all patient data were included in the analysis. The total QoR-15 scores for Group A and Group B at 24 hours were 134.8±6.65 and 128.9±6.12, respectively (*P*=0.006). The QoR-15 scores at 48 hours for groups A and B were 136.7±6.02 vs 132.10±6.8 (*P*=0.029), respectively. The pain score was lower in Group A than in Group B at rest and during movement, with *P*=0.015 and 0.001, respectively, and all the other secondary outcomes were comparable between the groups.

**Conclusion:**

Postoperative analgesia with continuous low-dose epidural morphine and ketamine via an intraoperatively placed epidural catheter provides superior QoR after major lumbar spine surgery as compared to intravenous morphine and ketamine.

**Main Points**

• Pain affects recovery; appropriate pain management enhances the quality of recovery (QoR), improving the quality of life.

• Most studies looked at the various modes of analgesia, their effect on acute and chronic pain, and the rescue analgesic requirements following major spine surgery. Literature regarding the impact of analgesia on QoR following major spine surgery is scarce. This study assessed the effect of analgesia on the QoR using the QoR-15 questionnaire following major spine surgery.

• Continuous administration of low-dose epidural morphine and ketamine analgesia via an intraoperatively placed epidural catheter without local anaesthetics provides excellent analgesia and superior QoR following major lumbar spine surgery compared to systemic morphine and ketamine.

## Introduction

The number of patients undergoing major spine surgery for degenerative disease, involving the lumbar spine, is constantly increasing worldwide.^1^ Pain after major spine surgery is moderate to severe during the first 48-72 hours.^2, 3, 4^ Appropriate pain management enhances recovery, reduces chronic pain, and improves the quality of life. Administration of systemic opioids is considered the mainstay for postoperative pain management; controlling pain with a high dose of systemic opioids often leads to excessive sedation, respiratory depression, postoperative nausea and vomiting (PONV), paralytic ileus and prolonged ambulation time, which can affect the quality of recovery (QoR).^5, 6^ Though postoperative pain is an essential component of the QoR, the assessment of pain outcomes alone does not describe the global dimensions of postoperative recovery.The effect of analgesia on QoR is understudied. This study compared the effects of epidural versus systemic opioids with ketamine on QoR following major spine surgery.

The QoR-40, QoR-15, and QoR-9 questionnaires are routinely used to assess QoR after anaesthesia and surgery.^7^ Among all the QoR Questionnaires, the QoR-15 is a simple, validated, equally extensive, yet more efficient evaluation of QoR.^8^ It assesses five domains: physical comfort, independence, psychological support, pain, and emotional state. The total score ranges from 0-150, with a high score indicating good QoR. Its use in spine surgery is limited, and most studies have utilized QoR-40 as it was done before the validation of QoR-15.^9, 10^

High-dose opioid administration after tissue injury and inflammation can upregulate the N-methyl-D-aspartate receptor (NMDA) in the dorsal horn of the spinal cord. It can lead to central sensitization, opioid-induced hyperalgesia and opioid tolerance.^11, 12^ Hence, the addition of ketamine (NMDA receptor antagonist) as an anaesthetic adjuvant helps to enhance analgesia while reducing the effects mentioned above.^13, 14^

In our institution, intravenous administration of opioids with ketamine (continuous infusion or via the patient control analgesia system), paracetamol (every 6^th^ hour) and non-steroidal anti-inflammatory drugs (every 8^th^ hour) was the standard of practice for pain management after major spine surgery till 2019. Hence, we wanted to compare intravenous morphine and ketamine with epidural morphine and ketamine on QoR using the QoR-15 questionnaire following major spine surgery. We felt that assessment of QoR is better than assessing only the pain to study the analgesic efficacy of a particular analgesic technique. We hypothesized that continuous low-dose epidural morphine and ketamine would provide superior QoR by providing adequate analgesia with minimal side effects compared to intravenous morphine and ketamine administered via a patient-controlled analgesia (PCA) system. The primary outcome was to study the impact of analgesia on QoR using the QoR-15 questionnaire 24 and 48 hours after major lumbar spine surgery. The secondary outcomes were the pain score at various time points, opioid requirement, rescue analgesic requirement, haemodynamics, ambulation time, time taken for solid intake, length of hospital stay, patient satisfaction, and the incidence of side effects of opioids and ketamine during the first 48 hours after surgery.

## Methods

This single-centre randomised controlled trial was conducted after obtaining approval from the Institutional Review Board of Christian Medical College, Vellore, India (approval no.: 12320, date: 30/10/2019) and registered with the Clinical Trial Registry of India (CTRI) (CTRI/2019/12/022513). Written informed consent was obtained from all recruited patients, and this study was conducted from January to December 2020 in accordance with the Declaration of Helsinki.

All patients with American Society of Anesthesiologists (ASA) grade 1-2, aged 18-70 years, with normal renal function who underwent transforaminal thoracolumbar/lumbar spine instrumentation, were recruited. Patients with ASA 3-4, body mass index>30 kg m^-2^, moderate-to-severe chronic obstructive pulmonary disease, impaired renal function (creatinine>1.4 mg dL^-1^), chronic liver disease, coagulation abnormalities, history of obstructive sleep apnea, allergy to study medications, psychiatric disorders, or chronic opioid use were excluded.

Patients scheduled for thoracolumbar/lumbar interbody fusion were screened, and eligible patients were randomised into group A (epidural morphine and ketamine) or group B (intravenous morphine and ketamine) using a computer-generated randomization sequence. Allocation concealment was performed using sealed, opaque envelopes. Randomization was done immediately before the start of anaesthesia. Patients were informed about the QoR score assessment details at the time of recruitment. QoR was assessed by the principal investigator, who was blinded to the study intervention. Patients were followed up till 48-hours regarding pain, rescue analgesic requirements, ambulation, and incidence of side effects of morphine and ketamine.

### Anaesthesia Protocol

On the day of surgery, after reassessment, patients were taken to the anaesthesia room, and an 18/16G intravenous cannula was placed. After placing an electrocardiogram, non-invasive blood pressure (BP), pulse oximetry, and end-tidal carbon dioxide, anaesthesia induction was carried out with fentanyl (2 mg kg^-1^), propofol (2 mg kg^-1^), ketamine (0.5 mg kg^-1^) and paralyzed using vecuronium (0.1 mg kg^-1^) and intubated with an appropriate size endotracheal tube. A 20/22G arterial cannula was placed to monitor the invasive arterial pressure after intubation. Anaesthesia was maintained with air, oxygen, and sevoflurane (0.8-1 minimum alveolar concentration) and titrated to maintain a bispectral index between 40-60. The mean arterial pressure was maintained within 15% of the baseline value during surgery. Morphine (0.1 mg kg^-1^), ketamine (0.05 mg kg^-1^ h^-1^) and paracetamol (20 mg kg^-1^) were administered for intraoperative analgesia, and the intraoperative pain response was treated with fentanyl (0.5 µg kg^-1^). Dexamethasone (4 mg) and Ondansetron (0.1 mg kg^-1^) were administered for PONV prophylaxis. Lignocaine (preservative-free) 1.5 mg kg^-1^ was administered to facilitate smooth extubation. Residual neuromuscular block (assessed using a train of four ratios) was reversed using neostigmine (0.05 mg kg^-1^) and glycopyrrolate (0.01 mg kg^-1^). Extubation was performed once the criteria were met. Postoperative analgesic regimens were followed according to the group allocation. Both groups received paracetamol (1 gm every 6^th^ hour) as part of a multimodal analgesia protocol. At any time point, if the numerical rating scale (NRS) score was >4, intravenous diclofenac (50 mg) was administered as a first rescue analgesic. If pain persists (NRS>4), tramadol (50 mg) and paracetamol (325 mg) combination was administered as a second rescue analgesia drug.

**Postoperative Analgesia Protocol for Group A:** At the end of instrumentation, after ensuring that there was no cerebrospinal fluid (CSF) leak at the surgical site, the surgeon placed a 20G epidural catheter under direct vision through the cranial end of the laminectomy defect, directed cranially up to 5-6 cm. After ensuring a negative CSF aspiration, 2 mL of 0.9% normal saline was injected. While injecting saline, the anaesthesiologist ensured no undue resistance, and the surgeon ensured that saline did not appear in the surgical field; both measures ruled out catheter malposition. The caudal end of the epidural catheter was taken out through the skin and secured away from the surgical incision site. Morphine (1 mg) and ketamine (10 mg) were administered as bolus through the catheter for over 5 minutes. Seven mg of morphine (0.7 mL) and 50 mg (1 mL) of ketamine were diluted in 0.9% normal saline (48.3 mL) and loaded in a 50 ml continuous analgesia drug delivery (CADD) pump, which was set at the rate of 1 mL hr^-1^ for 48 hours (morphine-0.14 mg, ketamine-1 mg hr^-1^). Infusion was initiated soon after extubation.

**Postoperative Analgesia Protocol for Group B:** Patients in Group B received intravenous morphine and ketamine through PCA using a CADD pump. Morphine (50 mg) and ketamine (50 mg) were diluted in 44 mL 0.9% normal saline at a concentration ratio 1:1 and loaded into a 50 mL CADD pump. Patients received a 0.015 mL kg^-1^bolus^-1^ of the study drug with a lockout period of 10 mins (six boluses hour^-1^), which was delivered intravenously for 48 hours.

### Data Analysis

Preoperatively, demographics, ASA status, presenting symptoms, duration of back pain and its severity, radicular pain, neurological deficits, and associated comorbidities were recorded. Intraoperatively, the levels of instrumentation, blood loss, crystalloid administration, fentanyl, propofol requirement, duration of surgery, and anaesthesia were documented. Postoperatively, the heart rate, BP, respiration, and sedation scores were noted regularly. Pain scores were measured using the NRS (0=No pain, 10=Worst pain) at rest and during movement at arrival and 2, 6, 12, 24, 36, and 48 hours after arrival to the ward (7-time points). The total dose of morphine consumed at 24 and 48 hours in the intravenous group was noted (Group B). The time taken to receive the first dose of rescue analgesia after arrival to the postoperative ward was noted, and the total doses of rescue analgesic and antiemetic administered to control pain and PONV, respectively, were recorded during the first 24 and 48 hours. The time to start moving in and around the bed without support was taken as the ambulation time, and the time of solid diet intake and the duration of hospital stay were noted. Patient satisfaction was assessed using a NRS (1-10) and graded as excellent (9-10), very good (7-8), good (5-6), fair (3-4), poor (1-2) and compared between the groups.

QoR was assessed using the QoR-15 questionnaire. The questionnaire consisted of 15 questions that examined ﬁve domains of patient recovery, such as physical comfort (questions 1-4,13), physical independence (questions 5,8), psychological support (questions-6,7), emotional state (questions-9,10,14,15) and pain (questions-11,12). Each question uses a Likert scale (0-10), with a total score ranging from 0-150. A higher score indicates good QoR. In the original questionnaire, we modified the 8^th^ question as “Able to move, turn around or sit up in the bed without support”. This study examined the total score and the score for the individual domains (five domains) at 24 and 48 hours after surgery and compared them between groups A and B.

Literature on QoR using QoR-15 following major spine surgery was unavailable while designing this study. Hence, the statistical input was taken from the article by Mariappan et al.^9^ The authors compared QoR using the QoR-40 score following anterior cervical discectomy and fusion in patients who received a superficial cervical plexus block versus those who received no block for perioperative analgesia. In this study, we used the QoR-15 instead of the QoR-40 questionnaire; hence, we had taken a six-point difference in the total QoR-15 score to show a clinically meaningful difference of 15%, with an alpha error of 5% and a 95% confidence interval. The required sample size was 15 in each group. With 15% dropouts, we decided to study approximately 20 subjects in each arm for 40 subjects.

### Statistical Analysis

Descriptive statistics are reported as mean and standard deviation for continuous variables (normally distributed) and as median and interquartile range (IQR) for skewed data. All categorical variables were reported as frequencies and percentages. The Shapiro-Wilk and Kolmogorov-Smirnov tests were used for normality. To compare the mean difference between groups, the Student’s and Independent Samples t-tests was used for normally distributed data, and the Mann-Whitney U test was used for data with skewed distribution. Chi-square and Fisher’s exact tests were used to determine the association between groups and categorical variables. A generalized estimating equation was used to compare the two groups over time for postoperative haemodynamics and sedation scores during the first 48 hours at specified intervals. All tests were two-sided at a significance level of α=0.05. All analyses were performed using SPSS software (version 21.0; IBM Corp, Armonk, NY, IBM Corp).

## Results

**Figure 1:**
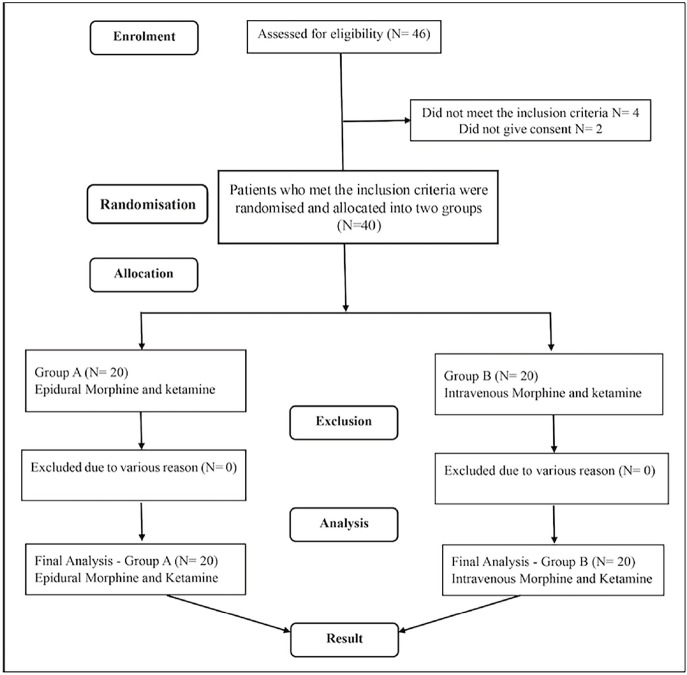
Consort diagram.

A total of 46 patients were screened, of whom 40 were recruited. Twenty patients received epidural morphine and ketamine (Group A), and another 20 received intravenous morphine with ketamine (Group B) for postoperative analgesia. All patients underwent lumbar instrumentations except four in Group A (epidural) and two in Group B (intravenous), who underwent thoracolumbar instrumentation involving T11 and below. The results were analysed using the intention-to-treat method; none were lost to follow-up. Figure 1 depicts a consort diagram. The demographics and baseline characteristics were comparable between the groups (Table 1). The intraoperative propofol and fentanyl requirements, blood loss, total intravenous fluid administration, duration of anaesthesia, surgery, and instrumentation levels were comparable between the groups (Table 2).

The total QoR-15 score at 24 hours for Group-A (epidural morphine and ketamine) and Group-B (intravenous morphine and ketamine) were 134.8±6.65 versus 128.9±6.12, respectively (*P*=0.006). The QoR scores at 48 hours for groups A and B were 136.7±6.02 versus 132.10±6.8 (*P*=0.029), respectively. The QoR-15 scores for the five subdomains were compared between groups. The subdomain scores of physical independence and emotional support were significantly higher in Group A at 24 hours. The pain and emotional support scores were significantly better in Group A at 48 hours. The other subdomain scores were comparable between the groups. The total QoR-15 scores and the scores for each domain are shown in Table 3.

**Figure 2:**
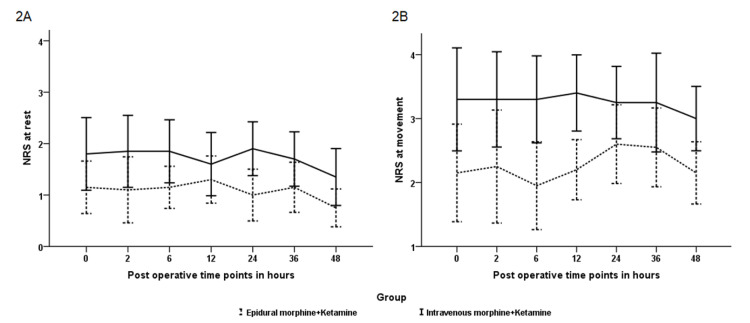
Numerical rating scale at rest (2A) and movement (2B) at various time points during the first 48 hours postoperative period. NRS, numerical rating scale.

The mean NRS ranged between 1-4 at most time points, both at rest and during movement, for both groups. A pain score of <4 at most time points, even during movement, signifies that both techniques provided good-quality analgesia following thoracolumbar/lumbar spine instrumentation and fusion. The pain score was significantly lower in group A (epidural) than in group B (intravenous), both at rest and during movement, at each time point (*P*=0.015 and 0.001, respectively) (Figure 2). The mean dose of morphine consumed during the first 24 hours in group B was 18.25±11.36 mg, and for 48 hours was 27.4±13.3 mg. Patients in group A received a fixed dose of 4.5 mg of epidural morphine at 24 hours and 8 mg at the end of 48 hours. The time taken to receive the first dose and the number of patients who received rescue analgesia during the first 24, 24-48 hours were comparable between the groups (Table 2). None of the patients in either group needed a second rescue analgesic drug.

**Figure 3:**
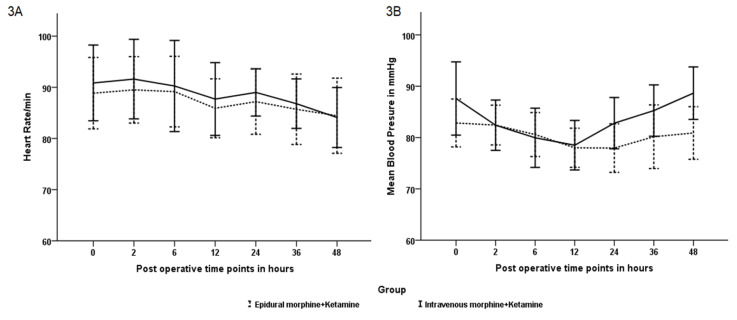
Heart rate (3A) and mean blood pressure (3B) at various time points during the first 48 hours postoperative period.

Postoperative haemodynamics (heart rate and BP) were comparable between the groups at all time points during the postoperative period. Figure 3 depicts the haemodynamics between the groups at various time points during the first 48 hours after surgery. The ambulation time was 15.41±4.11 hours for group A, while this was 12.56±5.14 hours for group B (*P*=0.061). The median time taken to resume their solid diet was 19.25 hours (IQR 25,75- 6.88, 21.83) for group A, while it was 14.78 (IQR 25,75- 4.18, 20.38) hours for group B (*P*=0.791). Both parameters were comparable between the groups. The duration of hospital stay was similar between the groups (Group A vs. B-5.00±2.44 vs. 5.15±1.81 days *P*=0.827).

There were 11/20 (55%) patients who had very good-to-excellent satisfaction in group A compared to 7/20 (35%) in group B (*P*=0.204). The proportions of patients who had fair satisfaction in groups A and B were 1/20 (5%) and 5/20 (25%), respectively (*P*=0.182). However, these differences were not statistically significant.

There were 3/20 (15%) patients in group A who had PONV, compared to 9/20 (45%) in group B (*P*=0.082). The proportion of patients with abdominal distention and constipation in groups A and B was 1/20 (5%) and 5/20 (25%), respectively (*P*=0.182). None of the patients in either group experienced excessive sedation, respiratory depression, constipation, nystagmus, hallucinations, or pruritus that required treatment. The urinary catheter was removed on the second postoperative day, after which none of them had urinary retention.

## Discussion

This single centre randomised controlled trial compared QoR between patients who received epidural versus intravenous morphine and ketamine for postoperative analgesia following thoracolumbar/lumbar spine instrumentation showed that epidural morphine and ketamine analgesia provided superior QoR as compared to intravenous morphine and ketamine at 24 hours. The pain scores at rest and movement were lower at all time points in the epidural group than in the intravenous group, indicating that epidural analgesia provided better pain relief than the intravenous group.

A study by Leslie et al.^10^ has shown that postoperative pain affects the QoR in neurosurgical patients. There are studies comparing the different modes of analgesia on pain outcomes following spine surgery.^1, 2, 4, 15, 16^ Studying the pain outcome alone does not describe the global dimensions of postoperative recovery. Hence, we decided to study the impact of different modes of analgesia on QoR following spine surgery. There are only a few studies available in this regard^9, 17^, and Kleif and Gögenur^18^ classified QoR into four categories: excellent (136-150), good (122-135), moderate (90-121), and poor (0-89), based on the total QoR-15 score. Our study showed that both analgesic techniques provided good QoR at 24 h and good-to-excellent QoR at 48 h after major spine surgery. None of the patients in this study had a moderate or poor QoR (score<120).

Myles et al.^19^ recommended that a difference of 6 points in the QoR-15 score is considered significant to show the clinically meaningful difference between the two interventions. Similarly, our study also showed a statistically significant difference of 6 points between the groups at 24 hours, confirming that epidural analgesia using continuous low-dose epidural morphine and ketamine improves QoR. The study results by Myles and Myles^19^ were published after the commencement of our research. Hence, we could not utilize this study results for the sample size calculation.

Continuous/patient-controlled administration of intravenous opioids with or without adjuvants or epidural analgesia using local anaesthetics with opioids is commonly utilised for postoperative analgesia following major lumbar spine surgery. A recent meta-analysis of randomised controlled trials^20^ compared the analgesic efficacy of epidural and intravenous analgesia in major spine surgeries. Our study results showed that epidural analgesia provided superior analgesia and better patient satisfaction than intravenous analgesia. This study results were similar to the meta-analysis.^20^ The NRS at different time points during the first 48 hours was significantly lower in the epidural group than in the intravenous group.

The epidural dose used in the current study was selected based on our pilot study and our previous study by Prabhakar et al.^21^ in that study, single-shot epidural morphine (4 mg) and ketamine (30 mg) were administered for postoperative analgesia following lumbar laminectomy±discectomy, which provided 23±3 hours of analgesia. Since the current study was a major spine instrumentation surgery, we administered 8 mg morphine and 60 mg ketamine epidurally as a continuous infusion for 48 hours.

In this study, morphine was administered as a low-dose infusion for 48 hours rather than an intermittent bolus to prevent infection and reduce the side effects. Studies have shown that continuous infusion of low-dose epidural morphine was associated with fewer side effects than intermittent epidural morphine bolus in cardiac and thoracic surgeries.^22, 23^ To the best of our knowledge, this study is the first to administer a continuous infusion of epidural morphine and ketamine without local anaesthetics for postoperative analgesia following major lumbar spine surgery, which makes it unique. A combination of local anaesthetics and opioids is widely used for epidural analgesia following spine surgery.^4, 24^ Although the addition of local anaesthetics to opioids has an additive effect, we did not add local anaesthetics because they can interfere with neurological assessment following spine surgery. Even a dilute concentration of local anaesthetics can cause hypotension due to sympathetic blockade, which can be detrimental following spine surgery.

We chose to add ketamine as an anaesthetic adjunct to both intravenous and epidural morphine because of its multimodal effects. The use of opioids after the tissue injury can cause upregulation and activation of NMDA receptors, which can lead to central sensitization, opioid-induced hyperalgesia, and opioid tolerance.^11, 12, 25, 26^

The addition of ketamine has been shown to reduce the above mechanisms, hence decreasing the opioid requirement and its related complications.^25, 26^

### Study Limitations

Although epidural analgesia improves QoR following lumbar spine surgery, this technique cannot be utilized for analgesia in patients who have undergone previous lumbar spine surgery. Inserting an epidural catheter through scar tissue is technically challenging and can create an occult CSF leak, which increases morbidity. In addition, this technique cannot be used in patients who develop intraoperative CSF leaks. This study compared continuous infusion of low-dose epidural morphine and ketamine with patient-controlled intermittent intravenous administration of morphine and ketamine, which resulted in an inequivalent dose that could have influenced the study results.

## Conclusion

Continuous administration of low-dose epidural morphine and ketamine via an intraoperatively placed epidural catheter provides superior QoR following major lumbar spine surgery as compared to systemic morphine and ketamine analgesia.

## Ethics

**Ethics Committee Approval:** Ethical approval was obtained from the Institutional Review Board of Christian Medical College, Vellore, India (approval no.: 12320, date: 30/10/2019).

**Informed Consent:** Written informed consent was obtained from all recruited patients.

## Figures and Tables

**Table 1. Demographics, Comorbidities, and Presenting Symptoms Between the Groups:** 

**Parameters/Groups**	**Group A** **Epidural morphine and ketamine (n = 20)**	**Group B** **Intravenous morphine and ketamine (n = 20)**	***P *value**
Age (yr); mean ± SD	47.05±11.93	48.10±11.61	0.779
Gender; n (%) Male Female	- 5 (45.5) 6 (54.5)	- 2 (16.7) 10 (83.3)	0.1930
Weight (kg); mean ± SD	64.90±9.21	64.05±9.24	0.772
Height (cm); mean ± SD	158.3±11.8	156.4±11	0.591
BMI (kg/m^2^); mean ± SD	26.3±3.26	25.9±2.58	0.685
Comorbidities; n (%) Type II DM Hypertension	- 5 (25) 4 (20)	- 6 (30) 9 (45)	- 1.000 0.091
Presenting symptoms; n (%) Back pain Radiating pain Bladder/bowel involvement Lower limb weakness	- 20 (100) 17 (85) 2 (10) 2 (10)	- 20 (100) 19 (95) 1 (5) 0 (0)	- - 0.605 1.000 0.487

BMI, body mass index; DM, diabetes mellitus; N, number; SD, standard deviation.

**Table 2. Intraoperative, Postoperative Parameters and Side Effects Between the Groups:** 

**Parameters/Groups**	**Group A** **Epidural morphine and ketamine (n = 20)**	**Group B** **Intravenous morphine and ketamine (n = 20)**	***P *value**
**Intraoperative parameters**			
Levels of instrumentation; n (%) Two levels Three to four levels Five levels	- 13 (65) 4 (20) 3 (15)	- 16 (80) 3 (15) 1 (5)	0.606
Requirement of fentanyl (mg); mean ± SD	218±58.5	213±78	0.811
Requirement of propofol (mg); mean ± SD	195.0±46.85	241.05±107	0.086
Blood loss (mL); median (IQR: 25,75)	550 (425,900)	500 (400,625)	0.329
Crystalloid (mL); mean ± SD	2482.50±1205	2022.50±599.26	0.135
Duration of anaesthesia (mins); mean ± SD	318.00±74.9	295.60±52.4	0.280
Duration of surgery (mins); mean ± SD	244.75±79.40	231.7±63.67	0.568
**Postoperative parameters**			
Time taken to receive the first dose of rescue analgesia (hours); median (IQR: 25,75)	0 (0, 1.75)	0 (0, 1.75)	0.984
Number of patients who received rescue analgesia - 0-24 hours post-surgery; n (%)	5/20 (25)	4/20 (20)	1.000
Number of patients who received rescue analgesia - 24-48 hours post-surgery; n (%)	4/20 (20)	3/20 (15)	1.000
Time for ambulation (hours); mean ± SD	15.41±4.11	12.56±5.14	0.061
Time for solid food intake (hours); median (IQR: 25,75)	19.25 (6.88, 21.83)	14.78 (4.18, 20.38)	0.791
Duration of hospital stay (days); mean ± SD	5.00±2.44	5.15±1.81	0.827
**Postoperative side effects**			
PONV; n (%) Abdominal distension; n (%)	3/20 (15) 1/20 (5)	9/20 (45) 5/20 (25)	0.082 0.182

IQR, interquartile range; N, number; PONV, postoperative nausea and vomiting; SD, standard deviation.

**Table 3. Quality of Recovery-15 Score: Total Score and Scores for Each Domain at 24 and 48 Hours after Surgery:** 

**Parameters/Groups**	**Group A** **Epidural morphine and ketamine (n = 20)**	**Group B** **Intravenous morphine and ketamine (n = 20)**	***P *value**
**QoR-15 - total score**			
Score at 24 hours	134.8±6.7	128.9±6.1	0.006^*^
Score at 48 hours	136.7±6.0	132.1±6.8	0.029^*^
**Subdomain score at 24 hours**			
Pain	17.9±1.3	17.25±1.2	0.115
Physical comfort	46.1±2.9	44.55±3.4	0.127
Physical independence	14.8±1.9	13.5±1.4	0.017^*^
Physical support	19.3±1.2	19.4±0.9	0.649
Emotional support	36.6±2.5	24.6±2.9	0.027^*^
**Subdomain score at 48 hours**			
Pain	18.1±1.3	17.2±1.5	0.036^*^
Physical comfort	46.7±3.0	45.2±4.0	0.186
Physical independence	15.7±1.3	15.4±1.3	0.550
Physical support	19.3±0.9	19.8±3.7	0.523
Emotional support	36.9±2.0	35.5±2.3	0.027^*^

QoR, quality of recovery; *Statistically significant.
